# A Resequencing-Based Ultradense Genetic Map of *Hericium erinaceus* for Anchoring Genome Sequences and Identifying Genetic Loci Associated With Monokaryon Growth

**DOI:** 10.3389/fmicb.2019.03129

**Published:** 2020-01-31

**Authors:** Wenbing Gong, Chunliang Xie, Yingjun Zhou, Zuohua Zhu, Yahui Wang, Yuande Peng

**Affiliations:** Institute of Bast Fiber Crops, Chinese Academy of Agricultural Sciences, Changsha, China

**Keywords:** *Hericium erinaceus*, resequencing, high-resolution bin map, quantitative trait loci, monokaryon growth

## Abstract

*Hericium erinaceus* has attracted tremendous interest owing to its compelling health-promoting properties. However, breeding of elite cultivars of *H. erinaceus* is hindered by the lack of a genetic and molecular toolbox. Here, we performed resequencing analysis of 127 F_1_ single-spore isolates and constructed the first high-resolution genetic map of *H. erinaceus*. With the use of recombination bins as markers, an ultradense genetic map consisting of 1,174 bins (including 37,082 single-nucleotide polymorphisms) was generated. This newly developed genetic map covered 1,096.5 cM, with an average bin spacing of 0.95 cM. High collinearity between genetic map and *H. erinaceus* genome assembly was revealed by aligning scaffolds to this genetic map using bin markers as anchors. The application of this newly developed genetic map in quantitative trait locus (QTL) mapping was also elucidated, and four QTLs for monokaryon growth were recovered. One QTL, *mgr1*, which contributes 12.1% of growth variations, was located near the mating type A (MAT-A) loci. Overall, this newly constructed high-resolution genetic map (or bin map) could be used as reference in future genetic, genomic, and breeding studies on *H. erinaceus*.

## Introduction

*Hericium erinaceus* (Bull.) Pers., also called lion’s mane or yamabushitake, is a well-known culinary and medicinal mushroom and has been historically used in traditional Chinese medicine (He et al., 2017). *H. erinaceus* produces numerous bioactive compounds, including polysaccharides, terpenoids, proteins, lectins, and phenols (Friedman, 2015). Over the past decade, *H. erinaceus* has been extensively documented and exhibited a broad range of physiological and health-promoting properties such as antiaging, antioxidant, anticancer, immunomodulatory, and anti-gastritis and anti-metabolic diseases (Friedman, 2015; Tzeng et al., 2018; Wu et al., 2018).

Despite its nutritional and medicinal benefits, the current understanding of the molecular biology and genetics of *H. erinaceus* is limited. Hence, the genetic improvement of mushroom yield and quality is hampered, thereby preventing breeding of elite cultivars and ultimately affecting the sustainable development of the *H. erinaceus* industry. Its abundance in bioactive compounds, high yield, good quality, and resistance to diseases are the main targets of breeding schemes in mushrooms, including *H. erinaceus* (Gong et al., 2018a). However, the genetic improvement of these traits would be a great challenge because most of the traits of interest are governed by QTLs. The identification of QTLs underlying these complex traits through genetic mapping could greatly facilitate marker-assisted selection and accelerate the breeding progress (Gao et al., 2018).

High-resolution genetic linkage maps are crucial for the accurate identification and characterization of QTLs and candidate genes (Han et al., 2016). In addition, high-density genetic maps have been proven to be useful for various applications in marker-assisted selection, genome organization analysis, detection of recombination, and comparative genomic analysis (Gao et al., 2018). To date, only a few dozen genetic maps have been constructed for several widely cultivated mushrooms, including *Agaricus bisporus* (Foulongne-Oriol et al., 2010), *Pleurotus* spp. (Larraya et al., 2000; Okuda et al., 2009, 2012; Im et al., 2016; Gao et al., 2018), *Lentinula edodes* (Gong et al., 2016), and *Auricularia auricula-judae* (Lu et al., 2017). Most of the genetic linkage maps of edible mushrooms were generated using a few 10s to 100s of PCR-based markers (Foulongne-Oriol, 2012; Gong et al., 2016). The low coverage of genetic markers across genomes has hindered the identification of trait-regulating QTLs and genes. In *H. erinaceus*, no genetic linkage map has been generated to date, thereby preventing the identification of genotype–phenotype associations.

The development of genetically segregating populations is a prerequisite to genetic linkage-based mapping. Biparental populations such as F_2_, backcrosses, doubled haploids, recombinant inbred lines, and near-isogenic lines are commonly used in genetic map construction and QTL mapping in crops (Xu et al., 2017). In mushrooms, the selection of appropriate mapping populations relies on their reproductive mode (Foulongne-Oriol, 2012). The key to genetic map construction is the accurate estimation of recombination frequency during meiosis (Meng et al., 2015). F_1_ progenies such as SSIs derived from products of meiosis are ideal populations for genetic mapping and are frequently used in mushrooms (Gong et al., 2016; Gao et al., 2018). For the size of mapping populations, it is rare to use more than 100 progenies derived from meiotic spores in mushrooms, which limits mapping accuracy (Foulongne-Oriol, 2012).

In the past, the construction of a genetic map was quite tedious and time-consuming owing to the sample-by-sample low-throughput genotyping of markers in a set of mapping individuals (Foulongne-Oriol, 2012). The emergence of next-generation sequencing has simplified genetic map construction using high-throughput genotyping-by-sequencing, which allows the simultaneous discovery and genotyping of SNPs across the genomes of mapping individuals (Galpaz et al., 2018; Paudel et al., 2018). Despite the increase in the number of edible mushrooms that have been sequenced or resequenced (Grigoriev et al., 2014), the application of genotyping-by-sequencing in mushroom genetic studies is rare. High-throughput genotyping strategies have only recently been utilized in rapid genotyping of mushrooms (Au et al., 2013; Seplyarskiy et al., 2014; Gao et al., 2018). In *H. erinaceus*, no application of genotyping-by-sequencing in genetic studies has been reported.

Herein, through *de novo* SNP discovery and genotyping by high-throughput resequencing analysis of 127 SSIs in a mapping population, we constructed the first ultra-high-density genetic linkage map of *H. erinaceus*. Then, genome assemblies and the genetic map were integrated. The application of this newly developed genetic map in QTL mapping was also elucidated. Our objectives were to (1) construct a high-resolution genetic map for further genetic research and (2) detect QTLs for monokaryon growth in *H. erinaceus.*

## Materials and Methods

### Fungal Strains and Mapping Population

*Hericium erinaceus* monokaryon strains CS-4 (CCTCC AF 2018025) and 911-4 (CCTCC AF 2018054) were obtained from protoclones of the dikaryon strains HeCS (CCTCC AF 2018028) and He911 (CCTCC AF 2018027). The hybrid strain HeC9 was generated by mating between CS-4 and 911-4. The spores of HeC9 were isolated using spine-based elution (Gong et al., 2018b). In detail, we randomly selected eight spines, where the basidiospores are located, from a single mature fruiting body of HeC9. The basidiospores on the eight spines were washed with sterile water and then cultured in malt extract agar medium (3% malt extract, 0.3% soya peptone, and 1.5% agar). After germination, monokaryotic SSIs were randomly selected and identified under a microscope on the basis of the absence of clamp connections. Among the identified monokaryons, a total of 127 F_1_ SSIs derived from the hybrid strain HeC9 were selected and used as mapping population. All *H. erinaceus* strains used in this study were deposited at the Institute of Bast Fiber Crops, Chinese Academy of Agricultural Sciences (Changsha, China).

### Single-Nucleotide Polymorphism Identification

The genome of parent monokaryon CS-4 was *de novo* sequenced using the Illumina and PacBio platform. The genome of CS-4 was initially assembled using PacBio long reads and further corrected using accurate Illumina short reads. Finally, a 41.2-Mb genome of *H. erinaceus*, which consisted of 52 scaffolds with an N50 of 2.1 Mb, was assembled and used as reference genome (NCBI BioProject PRJNA541374, accession number SZZO00000000). For high-throughput genotyping of individuals in the mapping population, all of the 127 SSIs were resequenced using the Illumina HiSeq X Ten system at Shanghai OE Biotech., Co., Ltd., The total genomic DNA of monokaryon 911-4 and 127 SSIs was extracted from freeze-dried mycelia using a TIANGEN plant genomic DNA kit by following the manufacturer’s protocol. The DNA concentration was adjusted to 20 ng/μl. The paired-end libraries with an insert size of 350 bp were constructed for the subsequent paired-end (PE150) sequencing. Monokaryon 911-4 was sequenced with 240-fold genome coverage, and all of the 127 SSIs were sequenced with at least 20-fold coverage in this study.

The raw paired-end reads of monokaryon 911-4 and 127 SSIs were trimmed to remove the adaptors and low-quality bases using fastp (Chen et al., 2018). The reads were filtered with a sliding window of size 4, with average Phred score scale of 20 within the window. The trimmed reads were mapped to the CS-4 genome using bwa-mem v 0.7-17 (Li, 2013) with default parameters. SAMtools (Li et al., 2009) was used to position sort the aligned reads, and Picard tools (version 2.18.17^1^) was used to remove duplicates according to the mapping coordinates. SNP calling was performed according to the best practice pipeline using the GATK v 3.8.1 (McKenna et al., 2010). Briefly, the variants were called for SSI by the HaplotypeCaller with default parameters. A joint genotyping step for comprehensive variations union was performed on the gVCF files. In the initial filtering step, the SNP filter expression was set as QD < 2.0| | MQ < 40.0| | FS > 60.0| | SOR > 3.0| | MQRankSum < −12.5| | ReadPosRankSum < −8.0. Because all the SSIs in mapping population were monokaryons, only bi-allelic homozygous SNPs with a minimum mapping depth of 8 were retained. SNPs that were missing in more than 30% of the progeny were also removed.

### Bin Map Construction and Anchoring of Scaffolds

To increase mapping efficiency, individual SNPs were combined into recombination bins as genetic markers (Huang et al., 2009). We first merged the adjacent SNPs with the same genotype within 500 bp into consolidated SNPs (designated as Tags) using the script “SNP_genotyper.py.” To generate a set of high-quality non-redundant markers for genetic map construction, the co-segregated SNPs (Tags) were combined into a recombination bin. Then, using the python script “SNP_cosegregation.py,” the best tag with the most complete genotypic information was selected, on behalf of all the SNPs in a given bin, for map construction. The two scripts “SNP_genotyper.py” and “SNP_cosegregation.py” were released by Qi et al. (2018)^2^. For each bin, the genotype identical to CS-4 was marked as “a,” whereas that to 911-4 was marked as “b.”

Thus, a set of bins was employed as markers to construct a bin map using Joinmap 4.0 software (Van Ooijen and Voorrips, 2006) with the HAP1 model and default settings. Bin markers were assigned into LGs at an independence test LOD score from 3.0 to 10.0. The Kosambi mapping function was used to estimate the map distances. Bin map orders were scrutinized manually and, when necessary, further adjusted. After that, the co-segregated SNPs were re-added into the bin map to get the final genetic map. The integration of genome assemblies and the genetic map was performed using mapped bins as anchors. By comparing mapped SNP positions on scaffolds and positions on the genetic map, scaffolds of the CS-4 genome were anchored on LGs. The LGs of the bin map were graphically exhibited using the MapChart (Voorrips, 2002). Alignment of the genetic map and genome scaffolds was graphically exhibited using Circos (Krzywinski et al., 2009).

### Quantitative Trait Locus Mapping of Monokaryon Growth

For all the 127 SSIs, mgrs were determined on the malt extract agar medium, which were calculated as the radial extension of each mycelial colony per day (Gong et al., 2014). All the SSIs were incubated at 25°C in the dark with three repetitions. The effect of genotype on mgrs was assessed by one-way ANOVA using the Statistical Package for the Social Sciences version 25 (SPSS, Inc., Chicago, IL, United States). QTLs for *mgr* were scanned by CIM in WinQTLCart 2.5 (Wang et al., 2012). One thousand permutation tests were conducted to obtain the threshold for significant QTLs (*p* < 0.05). QTL confidence interval was identified as a genomic range with 1 LOD drop from the LOD peak of the QTL. To verify the result of CIM mapping, the inclusive CIM (ICIM) was also employed using QTL IciMapping v4.0 (Li et al., 2007) with mapping parameters of 1 cM step and 0.001 probabilities in stepwise regression.

## Results

### Genotyping of Single-Spore Isolate Population and Bin Map Construction

A total of 10 Gb of clean reads were generated for parent monokaryon 911-4 (240-fold genome coverage). At least 1 Gb of clean reads was obtained for each SSI (at least 24-fold genome coverage), with > 85% of the bases higher than Q30 (Supplementary Table S1). For all 127 SSIs, more than 90% of the clean reads could be aligned to the CS-4 reference genome. For parent monokaryon 911-4, 85.5% of the clean reads could be aligned to the CS-4 genome (Supplementary Table S1). Over 0.9 million SNPs were called within this SSI population. After SNP filtering and bin calling, we obtained a total of 1,522 bins. Only bins with minor allele frequencies (MAFs) > 20% were kept for subsequent linkage analyses.

**FIGURE 1:**
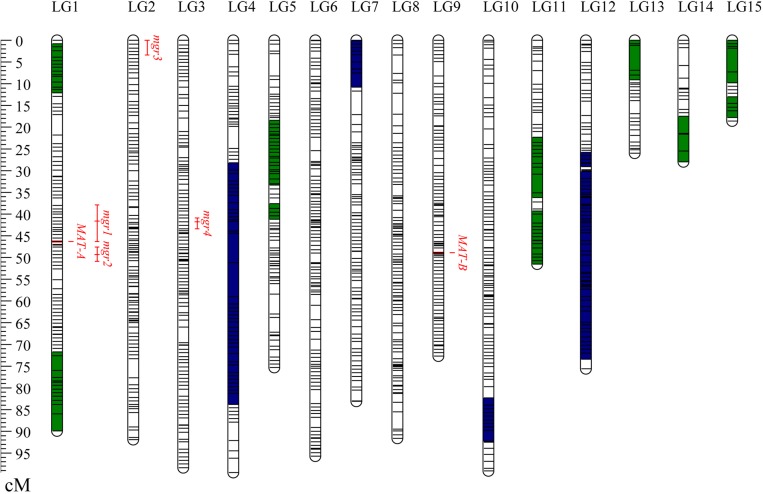
Distribution of bins on the genetic linkage map of *Hericium erinaceus.* The 15 LGs were randomly designated as LG1–LG15. Short lines in the LG bars indicate bin markers. The genetic position of bins is shown with a genetic ruler on the left. Segments in green representing the skewed bins in the segregation distortion regions are biased to CS-4, whereas segments in blue indicating the skewed bins are biased to 911-4. Four QTLs for mgr are shown on the right side of the LGs. The LOD-1 confidence interval is indicated by the length of the QTL bar, and the position of the LOD peak is represented by the short line in the middle of the QTL bar.

Finally, a total of 1,174 bins consisting of 37,082 consolidated SNPs were grouped into 15 LGs (Table 1). The total genetic length of the bin map was 1,096.5 cM and covered 31.1 Mb of the CS-4 genome. The average length of the bins was 26.5 kb, which was estimated as the ratio of covered physical lengths to the total number of mapped bins. In total, 50% of bin markers were < 5 kb in length, and there were three big bins larger than 1.0 Mb (Supplementary Figure S1). The number of bin markers per LG varied from 15 to 117, with an average of 78.3. The size of LGs ranged from 18.6 to 99.5 cM, with an average of 73.1 cM. The average interval of bins in most of LGs was less than 1.0 cM. No gap over 10 cM was found, and the maximum gap interval was detected on LG4 (7.86 cM). The distribution of bin markers on LGs is shown in Figure 1, and the genetic and physical positions of bins are presented in Supplementary Table S2. In the CS-4 genome, mating-type genes were identified by genome annotation and homology search. Mating-type loci A and B were mapped to scaffold6 and scaffold7, respectively. Accordingly, MAT-A and MAT-B were respectively mapped to LG1 (Tag_1981, 46.3 cM) and LG9 (Tag_28779, 48.9 cM) in the bin map (Figure 1). The correspondence between LG ID and chromosomes remained unresolved because the number of chromosomes in *H. erinaceus* is unknown.

**TABLE 1:** Characteristics of the genetic map of *Hericium erinaceus.*

**Linkage group**	**Mapped physical length (Mb)**	**Map length (cM)**	**No. of scaffolds**	**Recombination rate (cM/Mb)**	**No. of SNPs**	**No. of bins**	**Average length of bin (kb)**	**Average interval (cM)**	**Max interval (cM)**
LG1	4.2	89.9	2	21.3	5,839	104	40.4	0.87	4.65
LG2	2.0	91.9	2	46.0	2,649	108	18.5	0.86	4.35
LG3	3.5	98.4	1	27.9	4,882	115	30.4	0.86	3.65
LG4	3.2	99.5	1	31.2	1,322	77	41.6	1.31	7.86
LG5	2.2	75.3	3	34.5	2,258	81	27.2	0.94	5.13
LG6	3.1	95.7	2	31.4	4,585	117	26.5	0.83	3.34
LG7	1.9	83.1	2	43.5	2,419	88	21.6	0.95	5.38
LG8	2.5	91.2	1	36.6	2,497	98	25.5	0.94	4.81
LG9	2.1	72.7	2	34.8	2,566	98	21.4	0.75	3.31
LG10	3.4	99.1	2	29.4	4,257	101	33.7	0.99	3.56
LG11	0.7	51.5	1	78.0	905	46	15.2	1.14	4.30
LG12	1.7	75.6	1	45.3	2,038	85	20.0	0.90	3.32
LG13	0.3	26.0	1	92.9	328	24	12.5	1.13	5.70
LG14	0.2	28.0	1	140.0	291	17	11.8	1.75	4.08
LG15	0.3	18.6	2	74.4	246	15	20.0	1.33	5.50
Total	31.1	1,096.5	22.0^a^	-	37,082	1,174		15.56	68.94
Average	2.1	73.1	1.6	35.3^b^	2,472	78.3	26.5	0.95^c^	4.60

*SNP, single-nucleotide polymorphism; LG, linkage group. ^*a*^All the scaffolds were counted once. ^*b*^The average recombination rate in whole genome was calculated as the total genetic map size divided by the covered genome length. ^*c*^The average marker spacing over the whole map was calculated by dividing the total length of all LGs by the number of marker intervals (the number of mapped markers minus the number of LGs).*

**FIGURE 2:**
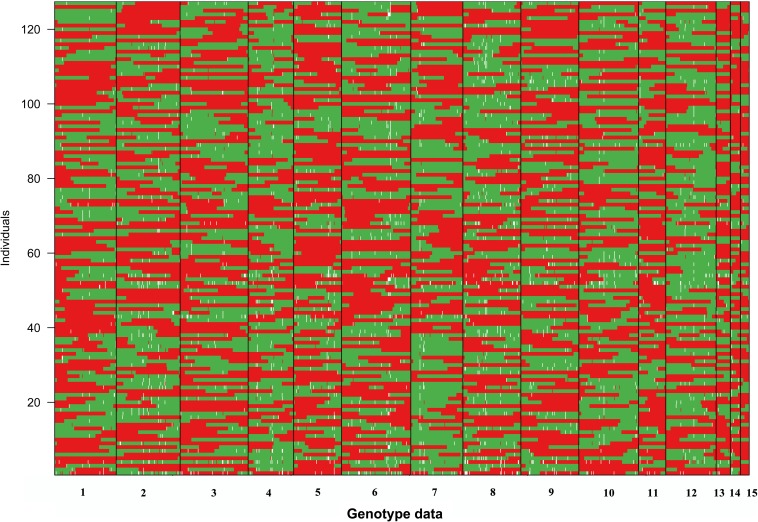
Visualization of the genotype of SSIs. The red segments originated from parent monokaryon CS-4, whereas the green segments are from 911 to 4. Missing data are shown in white.

Because limited crossovers occurred in the artificial biparental mapping population, for a given SSI, the large genome fragments from the same parent monokaryon were found (Figure 2). For all SSIs, no more than two crossovers occurred in LG1. In several SSIs such as 1-14 and 8-8, the whole segment of LG1 was inherited from CS-4, and no crossover was observed. The similar inheritance pattern was also detected in the other LGs (Figure 2). However, for all the 15 LGs, no SSI was inherited exclusively from a single parental line. Among all the SSIs, the minimal proportion of parental genotypes was 23.9% and 26.0%, which came from CS-4 and 911-4, respectively.

In this SSI population, 242 (20.7%) mapped bins were found to be skewed (*p* < 0.001), of which 110 bins were biased to the direction of parental line CS-4, whereas the remaining skewed bins were biased to 911-4. A total of 220 skewed bins (90.9%) were clustered as SDRs, which were defined as regions with three or more closely linked markers exhibiting segregation distortion (Paillard et al., 2003). A total of 15 SDRs, with 5–50 skewed bins per SDR, were distributed across 10 LGs (Table 2). All of the skewed bins in a given SDR skewed toward the same parent. The average genetic length of SDRs was 15.4 cM and varied from 3.6 to 55.6 cM.

**TABLE 2:** Segregation distortion regions detected in the genetic map of *Hericium erinaceus.*

**SDR**	**Linkage group**	**No. of skewed bins**	**Genetic length (cM)**	**Physical length (Mb)**	**Recombination rate (cM/Mb)**	**Distortion toward parent**
SDR1	1	14	11.3	0.44	25.7	CS-4
SDR2	1	14	18.2	0.19	95.8	CS-4
SDR3	4	42	55.6	2.64	21.1	911-4
SDR4	5	21	14.9	0.91	16.4	CS-4
SDR5	5	5	3.6	0.33	10.9	CS-4
SDR6	7	10	10.8	0.11	98.2	911-4
SDR7	10	10	10	0.14	71.4	911-4
SDR8	11	11	13.9	0.15	92.7	CS-4
SDR9	11	15	12.2	0.29	42.1	CS-4
SDR10	12	6	3.3	0.23	14.3	911-4
SDR11	12	50	43.2	0.88	49.1	911-4
SDR12	13	6	9.1	0.01	910.0	CS-4
SDR13	14	5	10.5	0.01	1,050.0	CS-4
SDR14	15	6	9.8	0.13	75.4	CS-4
SDR15	15	5	4.8	0.09	53.3	CS-4

### Alignment of Genome Assemblies to Genetic Map

**FIGURE 3:**
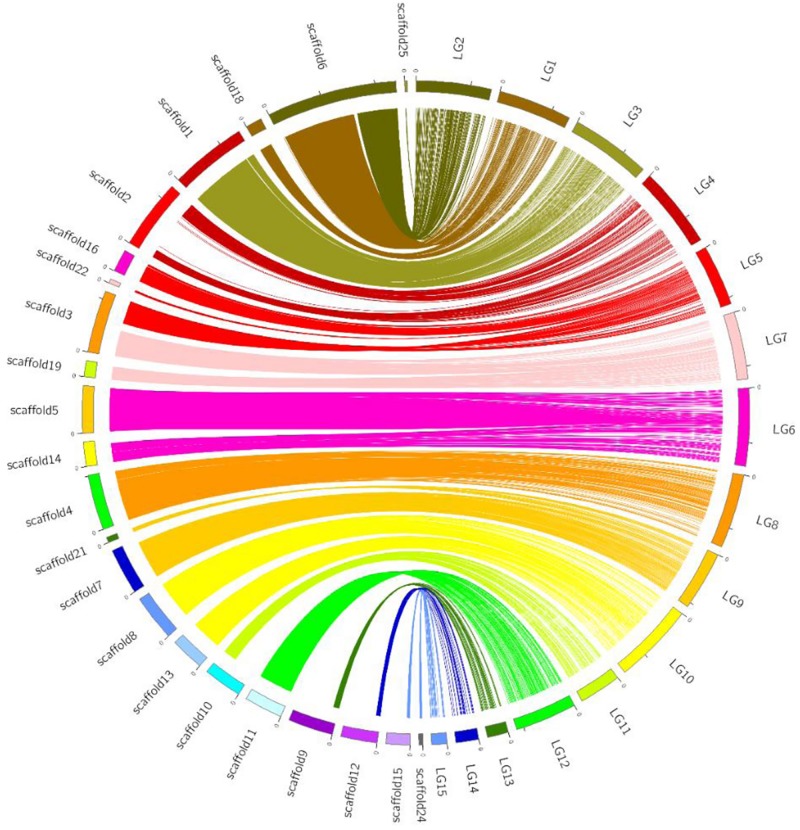
Graphical representation of the syntenic relationship between the physical and genetic maps of *H. erinaceus*. Linkage groups are depicted in colors at the right side of the circle, and the corresponding scaffolds are at the left side of the circle. Lines of the same colors connected the bins on LGs and physical positions on scaffolds.

Using bin markers as anchors, we aligned 22 scaffolds (total length: 38.8 Mb), representing 94.2% of the CS-4 genome, into 15 LGs of the genetic map. The schematic of assembled scaffolds-genetic map integration is shown in Figure 3. A high level of collinearity relationship between the physical sequence and genetic map of *H. erinaceus* was observed. An overwhelming majority of the marker orders on each LG were consistent with their position on the genome. Most of the scaffolds were mapped to a single LG, except for scaffold3 and scaffold6. Scaffold3 was dissected into two parts, corresponding to LG5 and LG7, whereas scaffold6 was divided into LG1 and LG2. Seven scaffolds corresponded to seven LGs (Table 1). A part of scaffold6 (∼3.8 Mb), gathered with scaffold18, was mapped to LG1, whereas the remainder of scaffold6, gathered with scaffold25, was anchored to LG2. In addition, LG5, LG6, LG7, LG9, LG10, and LG15 corresponded to two or three scaffolds (Supplementary Table S3).

The average recombination rate in *H. erinaceus*, calculated as the ratio of total genetic map size to covered physical lengths, was 35.3 cM/Mb. The recombination rates of different LGs varied from 21.3 to 140 cM/Mb. The recombination rate per LG was significantly negatively correlated with the physical length (*r*^2^ = 0.736, *p* < 0.001). The recombination rates of the three small LGs were relatively high. Compared with the other LGs, in LG1, the lowest recombination rate was observed (21.3 cM/Mb), where the MAT-A loci were located. To identify variations in recombination rates within chromosomes, the 37,082 consolidated SNPs were re-mapped into this genetic map. The SNPs in the same recombination bin had the same genetic position (Supplementary Table S4). We then divided the genome into non-overlapping 10-kb windows and calculated the recombination rate of the genome. The high level of variations in recombination rate within chromosomes was observed, with some regions entirely devoid of recombination and some regions exhibiting high recombination rates (Supplementary Figure S2). Recombination hotspot regions were defined as the chromosomal regions that presented an estimated recombination rate greater than a 10-fold increase in the genome-wide recombination rate (>350 cM/Mb) (Laurent et al., 2016). Accordingly, a total of 56 recombination hotspot regions were identified and unevenly distributed on the 15 LGs (Supplementary Figure S2 and Supplementary Table S5). The highest recombination rate (1,972.6 cM/Mb) was observed on LG8 (scaffold4: 30–31 kb), which was over 55-fold higher than the genome average recombination rate. In most cases, the higher recombination rates were observed in the LG ends, suggesting potential telomeric regions (Supplementary Figure S2). No crossover was detected in the 0.5-Mb region surrounding the MAT-A loci, suggesting a recombination coldspot. A normal recombination rate (37.4 cM/Mb) was observed in the region surrounding MAT-B loci. We also calculated the guanine–cytosine (GC) contents in the 56 recombination hotspot regions. Compared with that in the genomic background, the GC content was significantly higher in recombination hotspot regions (57.8% vs. 52.3%, Student’s *t*-test, *p* = 1.4 × 10^–21^).

### Quantitative Trait Locus Mapping for Monokaryon Growth

**FIGURE 4:**
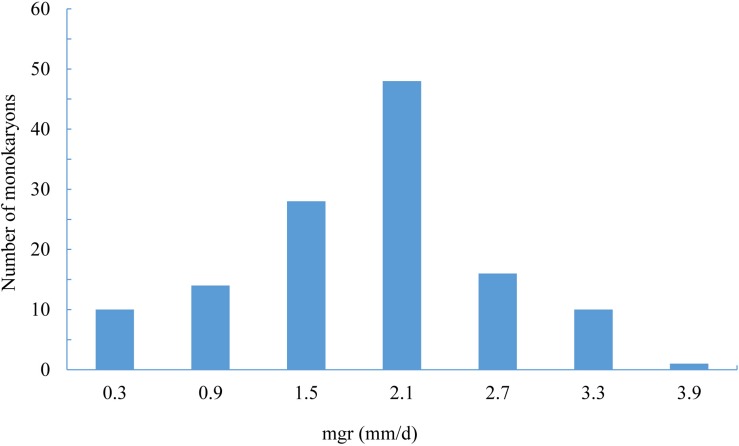
The distribution of monokaryon growth rates in SSI individuals.

The mgrs of the two parent monokaryons CS-4 and 911-4 were 3.2 and 1.29 mm/day, respectively. For the 127 SSIs, mgrs ranged from 0.33 to 4.27 mm/day, with an average of 1.89 mm/day (Figure 4). A significant effect of genotype on mgrs was revealed by one-way ANOVA (*p* < 0.01). The distribution of mgrs of SSIs derived from different spines is shown in Supplementary Figure S3. No significant difference was detected among growth rates of different spine-derived SSIs (*p* = 0.56). With the use of CIM, a total of four QTLs for mgrs were detected, of which two QTLs (*mgr1* and *mgr4*) were also verified by ICIM (Supplementary Figure S4). Together, the four QTLs accounted for 39.2% of mycelial growth variations, and the phenotypic variance explained by a single QTL ranged from 8.0% to 12.1% (Table 3). The physical length of the confidence intervals for these QTLs ranged from 52 to 2,467 kb. Both *mgr1* and *mgr2* imparted negative additive effects and were mapped to adjacent regions of LG1. Notably, *mgr1*, which was linked to Tag_5097, was mapped to the region encompassing 37.9 to 46.3 cM of LG1, where MAT-A was located. With a narrow confidence interval (40.9–43.4 cM, 52.3 kb) on LG4, *mgr4* was detected by CIM and ICIM and linked to bin marker Tag_12452. We then analyzed the genes in the confidence intervals of the four QTLs. Only 22 and 19 protein-coding genes were encompassed in the small physical intervals of *mgr3* and *mgr4*, respectively. However, 100s of genes were included in the confidence intervals of *mgr1* and *mgr2* on LG1. Several genes (such as those encoding homeodomain transcription factor, zinc finger transcription regulator, and glycosyltransferase), which had been reported to be possibly involved in vegetative growth and morphology of fungi, were localized within QTLs (Supplementary Table S6).

**TABLE 3:** Quantitative trait loci controlling the monokaryon growth rate of *Hericium erinaceus.*

**Locus**	**LG**	**Position^a^ (cM)**	**Nearest bin marker**	**LOD**	**Additive**	***R*^2^**	**CI^b^ (cM)**	**Length of CI (kb)**	**No. of genes in CI**	**ICIM**
*mgr1*	1	41.61	Tag_5097	4.98	−0.27	12.1	37.9–46.3	2,467.4	768	Y
*mgr2*	1	49.31	Tag_1625	3.73	−0.24	9.2	47.6–50.9	408.7	135	
*mgr3*	2	0.01	Tag_8350	3.39	−0.23	8.0	0–3.4	63.3	22	
*mgr4*	3	41.76	Tag_12452	4.16	0.25	9.9	40.9–43.4	52.3	19	Y

*Y: QTLs were also detected by inclusive composite interval mapping. QTLs, quantitative trait loci; LG, linkage group; LOD, likelihood of odds; ICIM, inclusive composite interval mapping. ^*a*^Position of the LOD score peak in cM. ^*b*^CI: confidence intervals supported by LOD-1.*

## Discussion

### Construction of a High-Dense Bin Map

High-resolution genetic linkage maps play indispensable roles in genetic, genomic, and breeding studies. To date, genetic studies on mushrooms are hindered by the fact that only a few genetic maps are currently available, and only a few 100 non-specific PCR-based markers have been explored (Foulongne-Oriol, 2012). Rapid advances in high-throughput sequencing have revolutionized SNP discovery and genotyping analyses (Hu et al., 2017). Here, by employing resequencing analysis of 127 SSIs in a mapping population, we constructed the first high-resolution genetic map of *H. erinaceus*. Because there are a relatively small number of recombination events in biparental mapping populations, high-throughput genotyping by sequencing-based approaches results in highly redundant markers per recombination bin (Jordan et al., 2018). Genotyping errors and missing data in these large datasets provide substantial challenges in map construction (Ronin et al., 2017). In this study, marker redundancy was reduced by calling bins that were defined by the recombination breakpoints on chromosomes. Then, the recombination bins were used in linkage map construction as the representative set of genetic markers. The recombination bin strategy for high-resolution genetic mapping has been extensively employed in plants (Han et al., 2016; Hu et al., 2017; Galpaz et al., 2018), but not in mushrooms. Here, a total of 37,082 consolidated SNPs that were grouped into 1,174 bins were mapped in the *H. erinaceus* genetic map. With the use of high-throughput SNP genotyping, the interval of markers reported here (<1.0 cM, no gap > 10 cM) was shorter than that of previously reported genetic maps in other mushrooms such as *A. bisporus* (Foulongne-Oriol et al., 2010) and *Pleurotus* spp. (Larraya et al., 2000; Im et al., 2016; Gao et al., 2018). This map also provides a reference for anchoring and sorting scaffolds during the assembly of the *H. erinaceus* genome.

The present genetic map covered 1,096.5 cM, spanning 15 LGs, of which three (LG13, LG14, and LG15) were relatively small. Currently, the number of chromosomes in *H. erinaceus* remains unclear. By microscopic observation, Zhou and Liu (1996) initially suggested that there were 12 chromosomes in *H. erinaceus*. The 12 large LGs may correspond to 12 chromosomes, and 3 small LGs correspond to small chromosome segments. Filtering of extremely distorted SNPs or bins during map construction may be one of the causes of fragmentation of a few chromosomes. Additional experiments such as contour-clamped homogeneous electric field gel analysis and high-throughput chromosome conformation capture analysis would provide more instructive clues on the actual number and conformation of *H. erinaceus* chromosomes.

Distorted segregation of markers has been commonly reported in linkage analysis (Manrique-Carpintero et al., 2016). The cluster distribution of skewed loci as SDRs has also been observed in other mushrooms, for example, *A. bisporus* (Foulongne-Oriol et al., 2010), *L. edodes* (Gong et al., 2014), and *P. eryngii* (Okuda et al., 2012). Several hypotheses such as the expression of lethal factors, biased selection of SSIs, and unbalanced selection of recognition loci have been used to explain distorted segregation of loci in fungi (Larraya et al., 2000; Foulongne-Oriol et al., 2010). No bias in segregation of bin markers surrounding the MAT-type loci was observed, suggesting that selection on the recognition loci could not be considered as the main cause of distorted segregation in this study. Most of the SDRs were relatively large with parent-specific segregation, suggesting that genetic factors influencing spore germination and growth are likely the causes for segregation distortion in this mapping population. One concern is that segregation distortion of markers may affect mapping quality (Hackett and Broadfoot, 2003). In this study, no differences in terms of mapping order due to distorted segregation were observed based on the high collinearity of the physical sequence and the genetic map.

### Alignment of Genome Assemblies and Genetic Map

The availability of a high-quality genome and genetic map greatly aids the identification of functional genes and genetic improvement of cultivars (Han et al., 2016). The high collinearity between sequence assemblies and the genetic map indicated that both the genetic map and genome assembly achieved high fidelity. Approximately, 94.2% (38.8 Mb, 22 scaffolds) of the CS-4 genome was anchored to the 15 LGs or pseudo-chromosomes. The unanchored scaffold, for example, scaffold20 (∼0.5 Mb), was enriched with repeated sequences. The majority of SNPs on this scaffold were heterozygous and thus were excluded from genetic mapping. In this study, a slight inconsistency of marker order on LGs and physical position on scaffolds was also observed, especially in regions with very low or no recombination (Supplementary Table S2), which was mainly caused by genotyping errors and missing data according to our in-house test.

Recombination plays a crucial role in the evolution of genomes, and extensive variations in recombination rates have been reported across sexually reproducing fungi (Laurent et al., 2016). In the button mushroom *A. bisporus* var. *bisporus*, genome-wide recombination suppression has been reported (∼11 cM/Mb), and meiotic crossovers were restricted to chromosome ends (Sonnenberg et al., 2016). The genome-wide recombination rate estimated in this study for *H. erinaceus* (35.3 cM/Mb) was higher than that of *A. bisporus* var. *bisporus* but was comparable with that of other mushrooms, for example, ∼32 cM/Mb in *P. ostreatus* (Larraya et al., 2000) and 31.6 cM/Mb in *P. tuoliensis* (Gao et al., 2018). Our results provided a comprehensive view of the recombination landscape in *H. erinaceus*. The recombination rate showed a highly uneven distribution among and along chromosomes, and recombination hotspot and coldspot regions were observed. The 56 recombination hotspot regions identified here could facilitate marker-assisted selection and accelerate the breeding progress by construction of populations with the higher recombination rate in specific genome regions. Suppressed recombination of the genomic regions involved in mating compatibility has been frequently reported in fungi (Idnurm et al., 2015). In *H. erinaceus*, an obvious recombination suppression or recombination coldspot was also found in the region surrounding the MAT-A loci. The increase in recombination rate in subtelomeric regions was observed in many species (Laurent et al., 2016). In this study, the higher recombination rates were observed for the three small LGs (LG13–LG15), which suggested that these were likely telomeres or subtelomeric regions of other chromosomes.

### Quantitative Trait Loci for Monokaryon Growth

One principal interest of genetic maps is to identify QTLs or genes for phenotypic traits of interest. The application of this newly developed genetic map of *H. erinaceus* in QTL mapping was also elucidated. Mycelial growth has attracted tremendous interest owing to its correlation with resistance to diseases (Larraya et al., 2002). Four QTLs for mycelial growth were detected and together contributed 39.2% of total mycelial growth variation, which indicated that mgrs were shaped by multiple small-effect loci. The QTL *mgr1* was localized near MAT-A. In some Basidiomycetes, an association of mycelial growth and sexual recognition has been observed (van der Nest et al., 2009; Sivolapova et al., 2012; Gong et al., 2014). The results of the present study in *H. erinaceus* have provided additional evidence that mycelial growth is influenced by sexual recognition loci. However, only part of the QTLs for mycelial growth was positioned near the recognition loci. There seemed to be a correlation without causal relation between mycelial growth and the sexual recognition system. The confidence interval of *mgr1* was significantly longer than the remaining three loci, which may be caused by the recombination suppression of regions surrounding the MAT-A loci. The low recombination rate (3.4 cM/Mb) of this confidence interval implied that it is arduous to narrow down the boundaries of the target loci and identify the underlying genes.

The integration of the genome assembly and genetic map allowed us to screen for putative candidate genes in QTL regions. Several genes, which have been reported to be possibly involved in vegetative growth and morphology of fungi, were found within the confidence intervals of these QTLs. For instance, several transcription factors such as homeodomain protein, MADS box transcription factor, and zinc finger protein were found within the confidence interval of *mgr1*. In *Schizophyllum commune*, transcription factors regulate various aspects of fruiting body formation, and the homeodomain protein Hom1 is involved in stimulating vegetative growth (Pelkmans et al., 2017; Vonk and Ohm, 2018). Similarly, in *Botrytis cinerea*, the homeobox *bchox8* gene was also found to be involved in vegetative growth and morphology (Antal et al., 2012). One C2H2-type zinc finger transcription regulator was found in the bin marker Tag_5097, which was located on the position of the LOD score peak of *mgr1*. In Tag_1625 (the peak position of *mgr2*), a DHHC-type zinc finger protein gene was also identified. In *S. commune* and *A. bisporus*, the Cys2His2 zinc finger protein gene *c2h2* has been shown to be involved in mushroom formation (Pelkmans et al., 2016). Two glycosyltransferase genes involved in glycosylphosphatidylinositol (GPI)-anchor biosynthesis were found in the peak positions of *mgr3* and *mgr4*. In fungi, GPI-anchored proteins often possess enzymatic activities that modify cell wall polymers and are indispensable for the continuous shape adaptation of the cell wall (De Groot et al., 2005). In *Candida albicans*, a glucosyltransferase coding gene *PHR1* plays a critical role in hyphal wall formation (Ragni et al., 2011). In *Ustilago maydis*, most of the genes encoding enzymes involved in the synthesis of the GPI anchor and secreted proteins were upregulated in the mycelium form compared with the yeast phase (Robledo-Briones and Ruiz-Herrera, 2013). Subsequent studies were required to further confirm the involvement of these genes in modifying vegetative growth. Nevertheless, the bin markers closely linked to these QTLs could be used in marker-assisted improvement. These putative candidate genes also provide clues in elucidating the molecular mechanism of vegetative growth in *H. erinaceus*.

## Conclusion

This study reported the utilization of the genotype-by-sequencing method for identification and genotyping of SNPs in *H. erinaceus*. The first high-resolution genetic map of *H. erinaceus* was generated using the genome-wide scale genotyping data of 127 SSIs. Then, by alignment of the scaffolds to LGs, high collinearity between the genome assembly and genetic map was revealed. In addition, four QTLs associated with mgr were also detected in the subsequent QTL mapping. The present study demonstrates the potential of using the newly developed genetic map to uncover the loci or genes regulating important agronomic and economic traits such as yield. Overall, this newly constructed high-resolution genetic map could be used as a reference in future genetic, genomic, and breeding studies.

## Data Availability Statement

The datasets generated for this study can be found in the NCBI BioProject: PRJNA541374, accession number: SZZO00000000.

## Author Contributions

WG conceived and designed the experiments, performed the experiments, analyzed the data, and wrote the manuscript. CX, YZ, and ZZ performed the experiments. YW analyzed the phenotypic and genotypic data. YP conceived and designed the experiments and reviewed drafts of the manuscript. All authors read and approved the final manuscript.

## Conflict of Interest

The authors declare that the research was conducted in the absence of any commercial or financial relationships that could be construed as a potential conflict of interest.

## Footnotes

1 http://broadinstitute.github.io/picard/

2 http://research.franklin.uga.edu/devoslab/scripts-used-gbs-pipeline

## Supplementary Material

FIGURE S1 The distribution of bin marker length.

FIGURE S2 The distribution of recombination rate in *Hericium erinaceus*. The recombination rates were estimated within non-overlapping 10-kb windows across the genome. The variations of recombination rates along LG1-LG12 are shown.

FIGURE S3 The box plot of monokaryon growth rate of SSIs derived from different spines.

FIGURE S4 Manhattan plot of QTLs for mgr. Based on the 1,000 permutation test, the LOD threshold for significant QTLs (*p* < 0.05) was 2.98, which was marked in a blue line.

TABLE S1 Re-sequence data of 127 SSIs and 911-4.

TABLE S2 Bin map information.

TABLE S3 Scaffolds coverage of linkage groups.

TABLE S4 The physical and genetic position of SNPs.

TABLE S5 The distribution of recombination hot regions.

TABLE S6 Genes in QTLs confidence intervals.

## Abbreviations

CIM: composite interval mapping
ICIM: inclusive composite interval mapping
LG: linkage group
LOD: likelihood of odds
MAT-A: mating type A
mgrs: monokaryon growth rates
QTL: quantitative trait locus
SDR: segregation distortion region
SNP: single-nucleotide polymorphism
SSIs: single-spore isolates.

## References

[B1] Antal Z. Rascle C. Cimerman A. Viaud M. Billon-Grand G. Choquer M. (2012). The homeobox BcHOX8 gene in *Botrytis cinerea* regulates vegetative growth and morphology. *PLoS One* 7:e48134. 10.1371/journal.pone.0048134 23133556 PMC3485016

[B2] Au C. H. Cheung M. K. Wong M. C. Chu A. K. Law P. T. Kwan H. S. (2013). Rapid genotyping by low-coverage resequencing to construct genetic linkage maps of fungi: a case study in *Lentinula edodes*. *BMC Res. Notes* 6:307. 10.1186/1756-0500-6-307 23915543 PMC3750829

[B3] Chen S. Zhou Y. Chen Y. Gu J. (2018). Fastp: an ultra-fast all-in-one FASTQ preprocessor. *Bioinformatics* 34 i884–i890. 10.1093/bioinformatics/bty560 30423086 PMC6129281

[B4] De Groot P. W. Ram A. F. Klis F. M. (2005). Features and functions of covalently linked proteins in fungal cell walls. *Fungal Genet. Biol.* 42 657–675. 10.1016/j.fgb.2005.04.002 15896991

[B5] Foulongne-Oriol M. (2012). Genetic linkage mapping in fungi: current state, applications, and future trends. *Appl. Microbiol. Biotechnol.* 95 891–904. 10.1007/s00253-012-4228-4 22743715

[B6] Foulongne-Oriol M. Spataro C. Cathalot V. Monllor S. Savoie J. M. (2010). An expanded genetic linkage map of an intervarietal *Agaricus bisporus* var. bisporus×A. *bisporus* var. *burnettii* hybrid based on AFLP, SSR and CAPS markers sheds light on the recombination behaviour of the species. *Fungal Genet. Biol.* 47 226–236. 10.1016/j.fgb.2009.12.003 20026415

[B7] Friedman M. (2015). Chemistry, nutrition, and health-promoting properties of *Hericium erinaceus* (Lion’s Mane) mushroom fruiting bodies and mycelia and their bioactive compounds. *J. Agric. Food. Chem.* 63 7108–7123. 10.1021/acs.jafc.5b02914 26244378

[B8] Galpaz N. Gonda I. Shem-Tov D. Barad O. Tzuri G. Lev S. (2018). Deciphering genetic factors that determine melon fruit-quality traits using RNA-Seq-based high-resolution QTL and eQTL mapping. *Plant J.* 94 169–191. 10.1111/tpj.13838 29385635

[B9] Gao W. Qu J. Zhang J. Sonnenberg A. Chen Q. Zhang Y. (2018). A genetic linkage map of *Pleurotus tuoliensis* integrated with physical mapping of the de novo sequenced genome and the mating type loci. *BMC Genom.* 19:18. 10.1186/s12864-017-4421-z 29304732 PMC5755439

[B10] Gong W. B. Li L. Zhou Y. Bian Y. B. Kwan H. S. Cheung M. K. (2016). Genetic dissection of fruiting body-related traits using quantitative trait loci mapping in *Lentinula edodes*. *Appl. Microbiol. Biotechnol.* 100 5437–5452. 10.1007/s00253-016-7347-5 26875873

[B11] Gong W. B. Li L. Zhou Y. Bian Y. B. Kwan H. S. Cheung M. T. (2018a). Detection of quantitative trait loci underlying yield-related traits in shiitake culinary-medicinal mushroom, *Lentinus edodes* (Agaricomycetes). *Int. J. Med. Mushrooms* 20 451–458. 10.1615/IntJMedMushrooms.2018026236 29953360

[B12] Gong W. B. Liu W. Lu Y. Y. Bian Y. B. Zhou Y. Kwan H. S. (2014). Constructing a new integrated genetic linkage map and mapping quantitative trait loci for vegetative mycelium growth rate in *Lentinula edodes*. *Fungal Biol.* 118 295–308. 10.1016/j.funbio.2014.01.001 24607353

[B13] Gong W. B. Xie C. L. Zhou Y. J. Zhu Z. H. Wang Y. H. Peng Y. D. (2018b). A simple and effective method of single spore isolation of *Hericium erinaceus*. *Edn. Med. Mushrooms* 26 363–366.

[B14] Grigoriev I. V. Nikitin R. Haridas S. Kuo A. Ohm R. Otillar R. (2014). MycoCosm portal: gearing up for 1000 fungal genomes. *Nucleic Acids Res.* 42 D699–D704. 10.1093/nar/gkt1183 24297253 PMC3965089

[B15] Hackett C. A. Broadfoot L. B. (2003). Effects of genotyping errors, missing values and segregation distortion in molecular marker data on the construction of linkage maps. *Heredity* 90 33–38. 10.1038/sj.hdy.6800173 12522423

[B16] Han K. Jeong H. J. Yang H. B. Kang S. M. Kwon J. K. Kim S. (2016). An ultra-high-density bin map facilitates high-throughput QTL mapping of horticultural traits in pepper (*Capsicum annuum*). *DNA Res.* 23 81–91. 10.1093/dnares/dsv038 26744365 PMC4833416

[B17] He X. Wang X. Fang J. Chang Y. Ning N. Guo H. (2017). Structures, biological activities, and industrial applications of the polysaccharides from *Hericium erinaceus* (Lion’s Mane) mushroom: a review. *Int. J. Biol. Macromol.* 97 228–237. 10.1016/j.ijbiomac.2017.01.040 28087447

[B18] Hu Z. Y. Deng G. C. Mou H. P. Xu Y. H. Chen L. Yang J. H. (2017). A re-sequencing-based ultra-dense genetic map reveals a gummy stem blight resistance-associated gene in *Cucumis melo*. *DNA Res.* 25 1–10. 10.1093/dnares/dsx033 28985339 PMC5824858

[B19] Huang X. Feng Q. Qian Q. Zhao Q. Wang L. Wang A. (2009). High-throughput genotyping by whole-genome resequencing. *Genom. Res.* 19 1068–1076. 10.1101/gr.089516.108 19420380 PMC2694477

[B20] Idnurm A. Hood M. E. Johannesson H. Giraud T. (2015). Contrasted patterns in mating-type chromosomes in fungi: hotspots versus coldspots of recombination. *Fungal Biol. Rev.* 29 220–229. 10.1016/j.fbr.2015.06.001 26688691 PMC4680991

[B21] Im C. K. Park Y. H. Hammel K. E. Park B. Y. Kwon S. W. Ryu H. J. (2016). Construction of a genetic linkage map and analysis of quantitative trait loci associated with the agronomically important traits of *Pleurotus eryngii*. *Fungal Genet. Biol.* 9 50–64. 10.1016/j.fgb.2016.05.002 27166667

[B22] Jordan K. W. Wang S. He F. Chao S. Lun Y. Paux E. (2018). The genetic architecture of genome-wide recombination rate variation in allopolyploid wheat revealed by nested association mapping. *Plant J.* 95 1039–1054. 10.1111/tpj.14009 29952048 PMC6174997

[B23] Krzywinski M. Schein J. Birol I. Connors J. Gascoyne R. Horsman D. (2009). Circos: an information aesthetic for comparative genomics. *Genom. Res.* 19 1639–1645. 10.1101/gr.092759.109 19541911 PMC2752132

[B24] Larraya L. M. Idareta E. Arana D. Ritter E. Pisabarro A. G. Ramírez L. (2002). Quantitative trait loci controlling vegetative growth rate in the edible basidiomycete *Pleurotus ostreatus*. *Appl. Environ. Microbiol.* 68 1109–1114. 10.1128/aem.68.3.1109-1114.2002 11872457 PMC123780

[B25] Larraya L. M. Perez G. Ritter E. Pisabarro A. G. Ramírez L. (2000). Genetic linkage map of the edible basidiomycete *Pleurotus ostreatus*. *Appl. Environ. Microbiol.* 66 5290–5300. 10.1128/aem.66.12.5290-5300.2000 11097904 PMC92458

[B26] Laurent B. Palaiokostas C. Spataro C. Moinaro M. Zehraoui E. Houston R. D. (2016). High-resolution mapping of the recombination landscape of the phytopathogen *Fusarium graminearum* suggests two speed genome evolution. *Mol. Plant Pathol.* 19 341–354. 10.1111/mpp.12524 27998012 PMC6638080

[B27] Li H. (2013). Aligning sequence reads, clone sequences and assembly contigs with BWA-MEM. *arXiv* [Preprint].

[B28] Li H. Handsaker B. Wysoker A. Fennell T. Ruan J. Homer N. (2009). The sequence alignment/map format and SAMtools. *Bioinformatics* 25 2078–2079. 10.1093/bioinformatics/ btp352 19505943 PMC2723002

[B29] Li H. Ye G. Wang J. (2007). A modified algorithm for the improvement of composite interval mapping. *Genetics* 175 361–374. 10.1534/genetics.106.066811 17110476 PMC1775001

[B30] Lu L. X. Yao F. J. Wang P. Fang M. Zhang Y. M. Zhang W. T. (2017). Construction of a genetic linkage map and QTL mapping of agronomic traits in *Auricularia auricula-judae*. *J. Microbiol.* 55 792–799. 10.1007/s12275-017-7241-6 28956350

[B31] Manrique-Carpintero N. C. Coombs J. J. Veilleux R. E. Buell C. R. Douches D. S. (2016). Comparative analysis of regions with distorted segregation in three diploid populations of potato. *G3* 6 2617–2628. 10.1534/g3.116.030031 27342736 PMC4978915

[B32] McKenna A. Hanna M. Banks E. Sivachenko A. Cibulskis K. Kernytsky A. (2010). The genome analysis toolkit:a MapReduce framework for analyzing next-generation DNA sequencing data. *Genom. Res.* 20 1297–1303. 10.1101/gr.107524.110 20644199 PMC2928508

[B33] Meng L. Li L. Zhang L. Wang J. (2015). QTL IciMapping: integrated software for genetic linkage map construction and quantitative trait locus mapping in bi-parental populations. *Crop. J.* 33 269–283. 10.1016/j.cj.2015.01.001

[B34] Okuda Y. Murakami S. Matsumoto T. (2009). A genetic linkage map of *Pleurotus pulmonarius* based on AFLP markers, and localization of the gene region for the sporeless mutation. *Genome* 52 438–446. 10.1139/g09-021 19448724

[B35] Okuda Y. Ueda J. Obatake Y. Murakami S. Fukumasa Y. Matsumoto T. (2012). Construction of a genetic linkage map based on amplified fragment length polymorphism markers and development of sequence-tagged site markers for marker assisted selection of the sporeless trait in the oyster mushroom (*Pleurotus eryngii*). *Appl. Environ. Microbiol.* 78 1496–1504. 10.1128/AEM.07052-11 22210222 PMC3294488

[B36] Paillard S. Schnurbusch T. Winzeler M. Messmer M. Sourdille P. Abderhalden O. (2003). An integrative genetic linkage map of winter wheat (*Triticum aestivum* L.). *Theor. Appl. Genet.* 107 1235–1242. 10.1007/s00122-003-1361-6 12898031

[B37] Paudel D. Kannan B. Yang X. Harris-Shultz K. Thudi M. Varshney R. K. (2018). Surveying the genome and constructing a high-density genetic map of napiergrass (*Cenchrus purpureus Schumach*). *Sci. Rep.* 8:14419. 10.1038/s41598-018-32674-x 30258215 PMC6158254

[B38] Pelkmans J. F. Patil M. B. Gehrmann T. Reinders M. J. Wösten H. A. Lugones L. G. (2017). Transcription factors of *Schizophyllum commune* involved in mushroom formation and modulation of vegetative growth. *Sci. Rep.* 7:310. 10.1038/s41598-017-00483-3 28331193 PMC5428507

[B39] Pelkmans J. F. Vos A. M. Scholtmeijer K. Hendrix E. Baars J. J. Gehrmann T. (2016). The transcriptional regulator *c2h2* accelerates mushroom formation in *Agaricus bisporus*. *Appl. Microbiol. Biotechnol.* 100 7151–7159. 10.1007/s00253-016-7574-9 27207144 PMC4947489

[B40] Qi P. Gimode D. Saha D. Schröder S. Chakraborty D. Wang X. (2018). UGbS-Flex, a novel bioinformatics pipeline for imputation-free SNP discovery in polyploids without a reference genome: finger millet as a case study. *BMC Plant Biol.* 18:117. 10.1186/s12870-018-1316-3 29902967 PMC6003085

[B41] Ragni E. Calderon J. Fascio U. Sipiczki M. Fonzi W. A. Popolo L. (2011). Phr1p, a glycosylphosphatidylinositol-anchored β (1,3)-glucanosyltransferase critical for hyphal wall formation, localizes to the apical growth sites and septa in *Candida albicans*. *Fungal Genet. Biol.* 48 793–805. 10.1016/j.fgb.2011.05.003 21601645

[B42] Robledo-Briones M. Ruiz-Herrera J. (2013). Regulation of genes involved in cell wall synthesis and structure during *Ustilago maydis* dimorphism. *FEMS Yeast Res.* 13 74–84. 10.1111/1567-1364.12011 23167842

[B43] Ronin Y. I. Mester D. I. Minkov D. G. Akhunov E. Korol A. B. (2017). Building ultra-high density linkage maps based on efficient filtering of trustable markers. *Genetics* 206 1285–1295. 10.1534/genetics.116.197491 28512186 PMC5500130

[B44] Seplyarskiy V. B. Logacheva M. D. Penin A. A. Baranova M. A. Leushkin E. V. Demidenko N. V. (2014). Crossing-over in a hypervariable species preferentially occurs in regions of high local similarity. *Mol. Biol. Evol.* 31 3016–3025. 10.1093/molbev/msu242 25135947 PMC4209137

[B45] Sivolapova A. B. Shnyreva A. V. Sonnenberg A. Baars I. (2012). DNA marking of some quantitative trait loci in the cultivated edible mushroom *Pleurotus ostreatus* (Fr.) *Kumm*. *Russian J. Genet.* 48 383–389. 10.1134/s1022795412040114 22730765

[B46] Sonnenberg A. S. Gao W. Lavrijssen B. Hendrickx P. Sedaghat-Tellgerd N. Foulongne-Oriol M. (2016). A detailed analysis of the recombination landscape of the button mushroom *Agaricus bisporus* var. *bisporus*. *Fungal Genet. Biol.* 93 35–45. 10.1016/j.fgb.2016.06.001 27288752

[B47] Tzeng T. T. Chen C. C. Chen C. C. Tsay H. J. Lee L. Y. Chen W. P. (2018). The cyanthin diterpenoid and sesterterpene constituents of *Hericium erinaceus* mycelium ameliorate Alzheimer’s disease-related pathologies in APP/PS1 transgenic mice. *Int. J. Mol. Sci.* 19 E598. 10.3390/ijms19020598 29463001 PMC5855820

[B48] van der Nest M. A. Slippers B. Steenkamp E. T. De Vos L. Van Zyl K. Stenlid J. (2009). Genetic linkage map for *Amylostereum areolatum* reveals an association between vegetative growth and sexual and self-recognition. *Fungal Genet. Biol.* 46 632–641. 10.1016/j.fgb.2009.06.002 19523529

[B49] Van Ooijen J. Voorrips R. (2006). *JoinMap 4.0. Software for the Calculation of Genetic Linkage Maps in Experimental Populations.* Wageningen: Kyazma Publisher.

[B50] Vonk P. J. Ohm R. A. (2018). The role of homeodomain transcription factors in fungal development. *Fungal Biol. Rev.* 32 219–230. 10.1016/j.fbr.2018.04.002

[B51] Voorrips R. E. (2002). MapChart: software for the graphical presentation of linkage maps and QTLs. *J. Hered.* 93 77–78. 10.1093/jhered/93.1.77 12011185

[B52] Wang S. Bastern C. J. Zeng Z. B. (2012). *Windows QTL Cartographer 2.5. Department of Statistics.* Raleigh, NC: North Carolina State University.

[B53] Wu F. Zhou C. Zhou D. Ou S. Zhang X. Huang H. (2018). Structure characterization of a novel polysaccharide from *Hericium erinaceus* fruiting bodies and its immunomodulatory activities. *Food Funct.* 9 294–306. 10.1039/c7fo01389b 29168863

[B54] Xu Y. Li P. Yang Z. Xu C. (2017). Genetic mapping of quantitative trait loci in crops. *Crop. J.* 5 175–184. 10.1016/j.cj.2016.06.003

[B55] Zhou Z. Y. Liu H. S. (1996). Study on the chromosome number of *Hericium erinaceus*. *J. Shenyang Norm. Univ.* 14 58–59. 10.1016/j.toxrep.2014.11.009 28962329 PMC5598247

