# Supplementary material for: A Resequencing-Based Ultradense Genetic Map of Hericium erinaceus for Anchoring Genome Sequences and Identifying Genetic Loci Associated With Monokaryon Growth
Source: Front Microbiol. 2020 Jan 31;10:3129. doi: 10.3389/fmicb.2019.03129 (PMC7005679; doi:10.3389/fmicb.2019.03129)
Supplement: FIGURE S1 — The distribution of bin marker length. [file Data_Sheet_1.pdf]

Supplementary Material

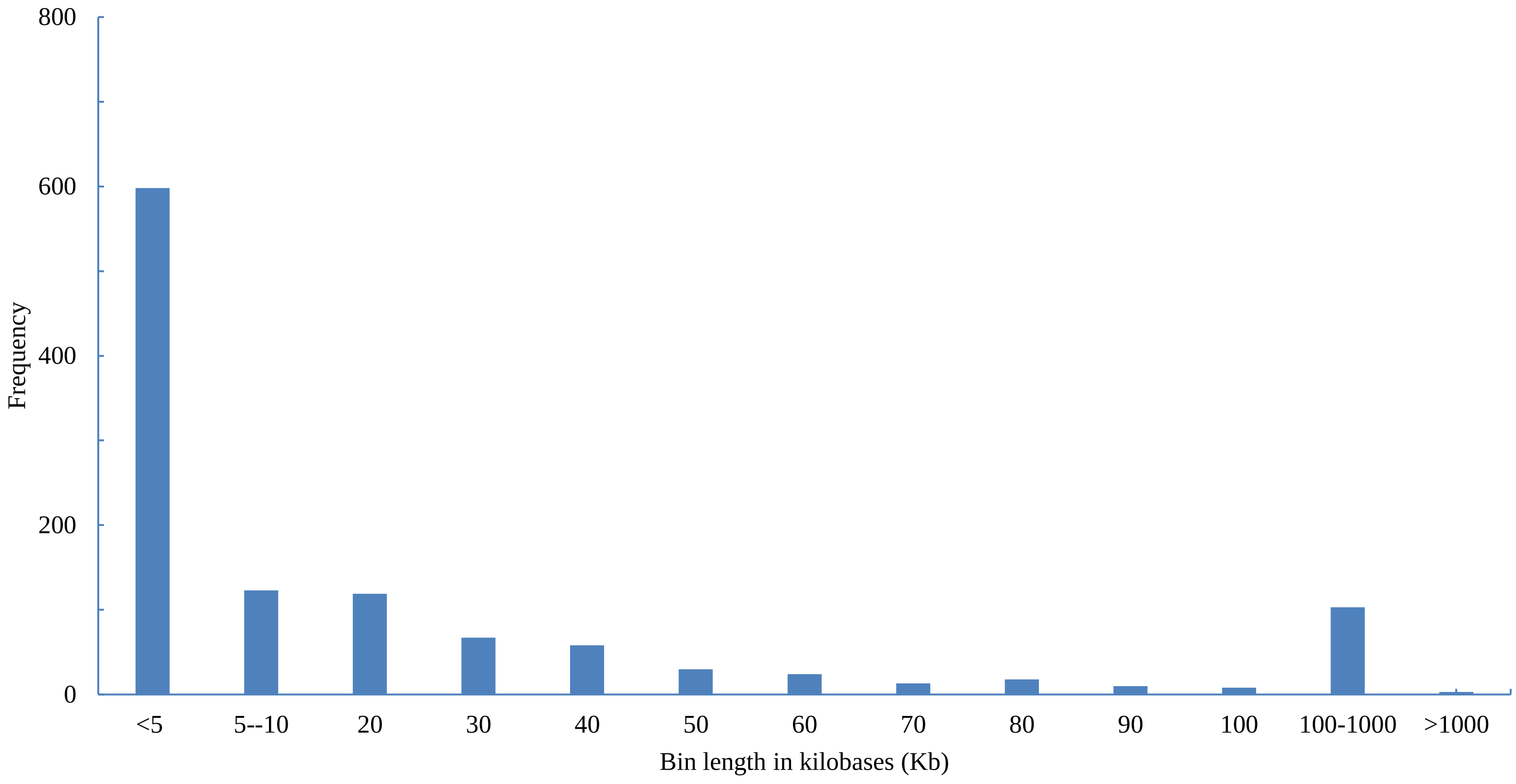

Figure S1. The distribution of bin marker length

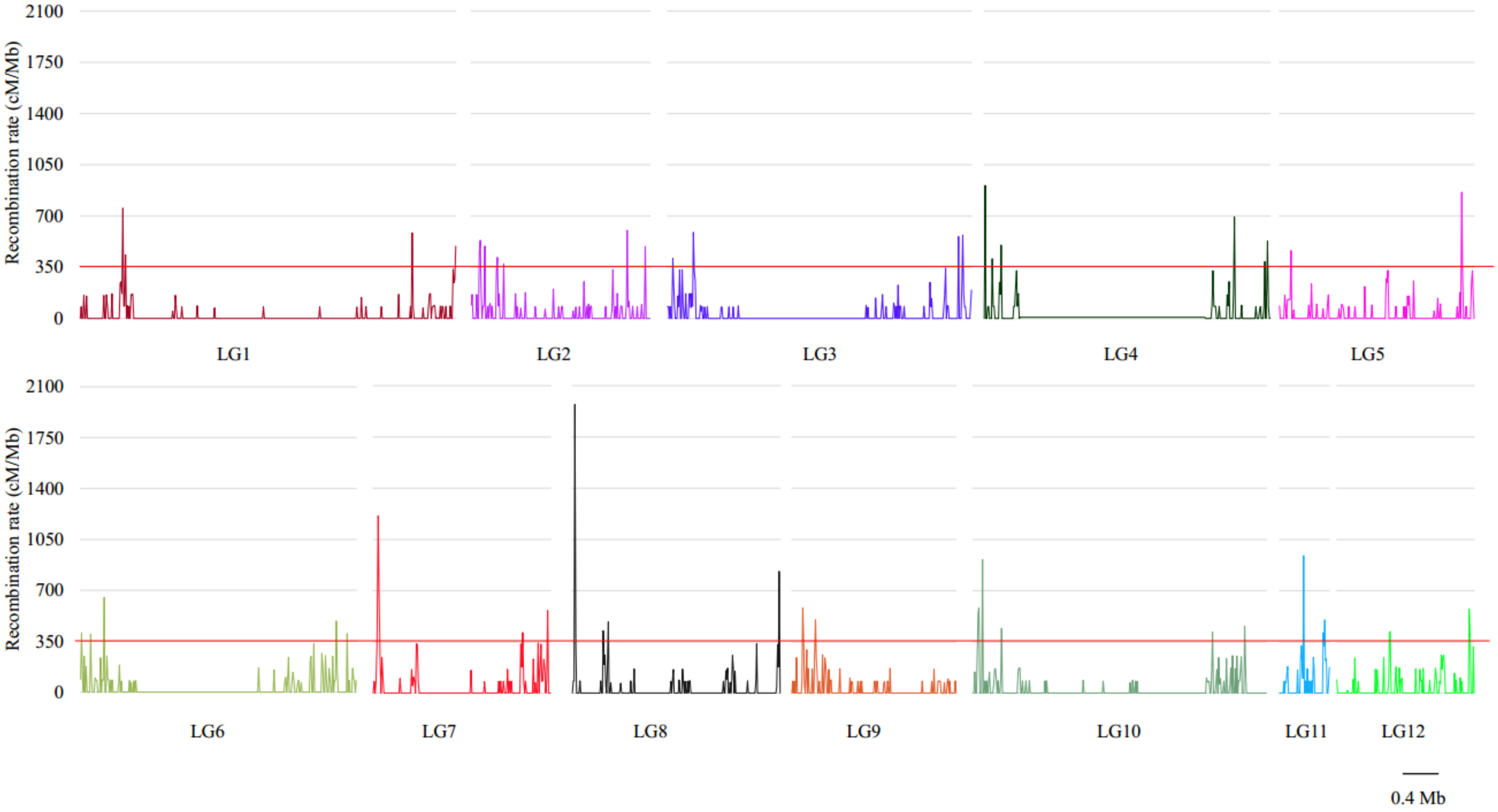

Figure S2. The distribution of recombination rate in *Hericium erinaceus*. The recombination rates were estimated within non-overlapping 10-kb windows across the genome. The variations of recombination rates along LG1-LG12 are shown.

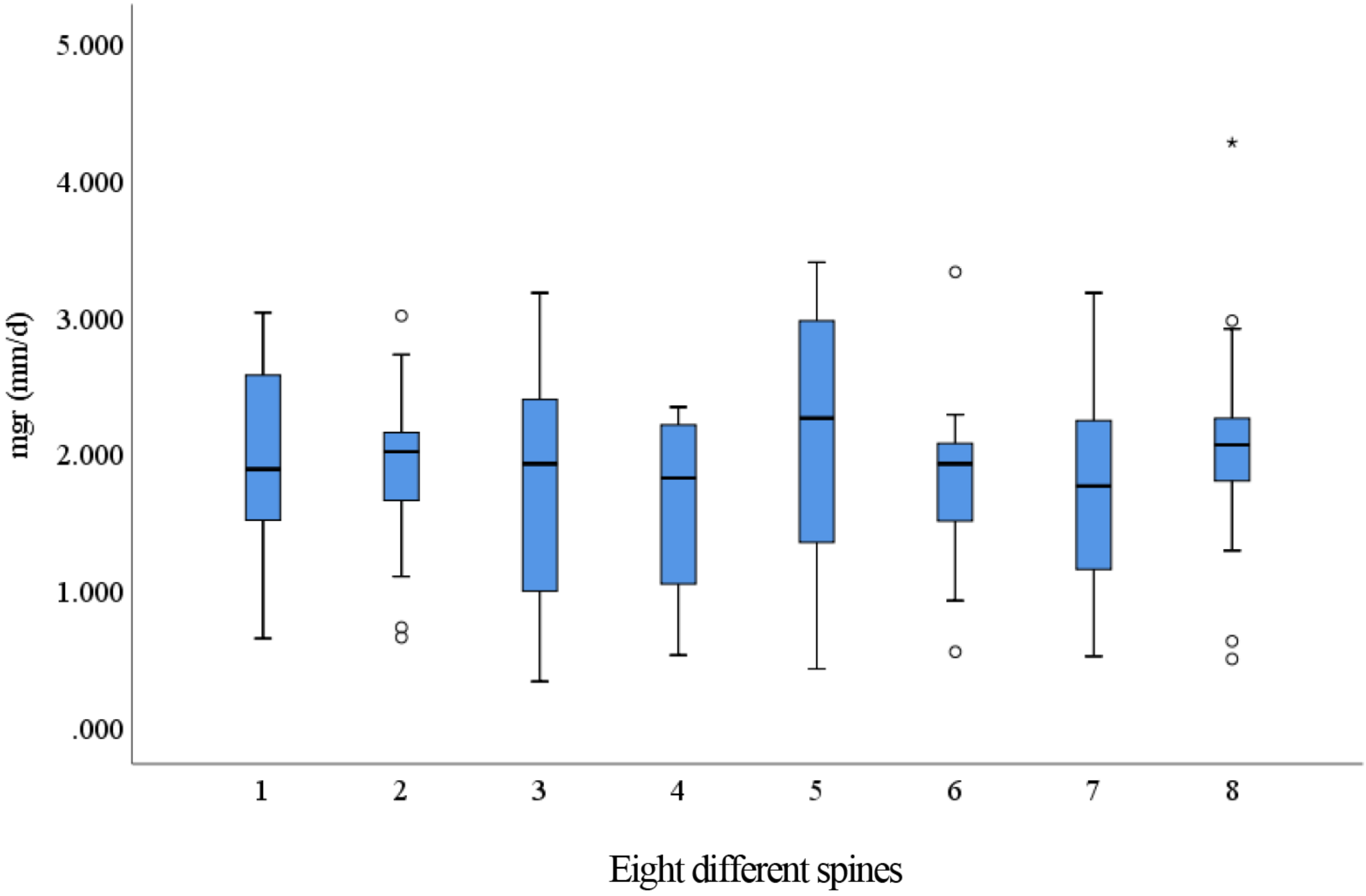

Figure S3. The box plot of monokaryon growth rate of SSIs derived from different spines.

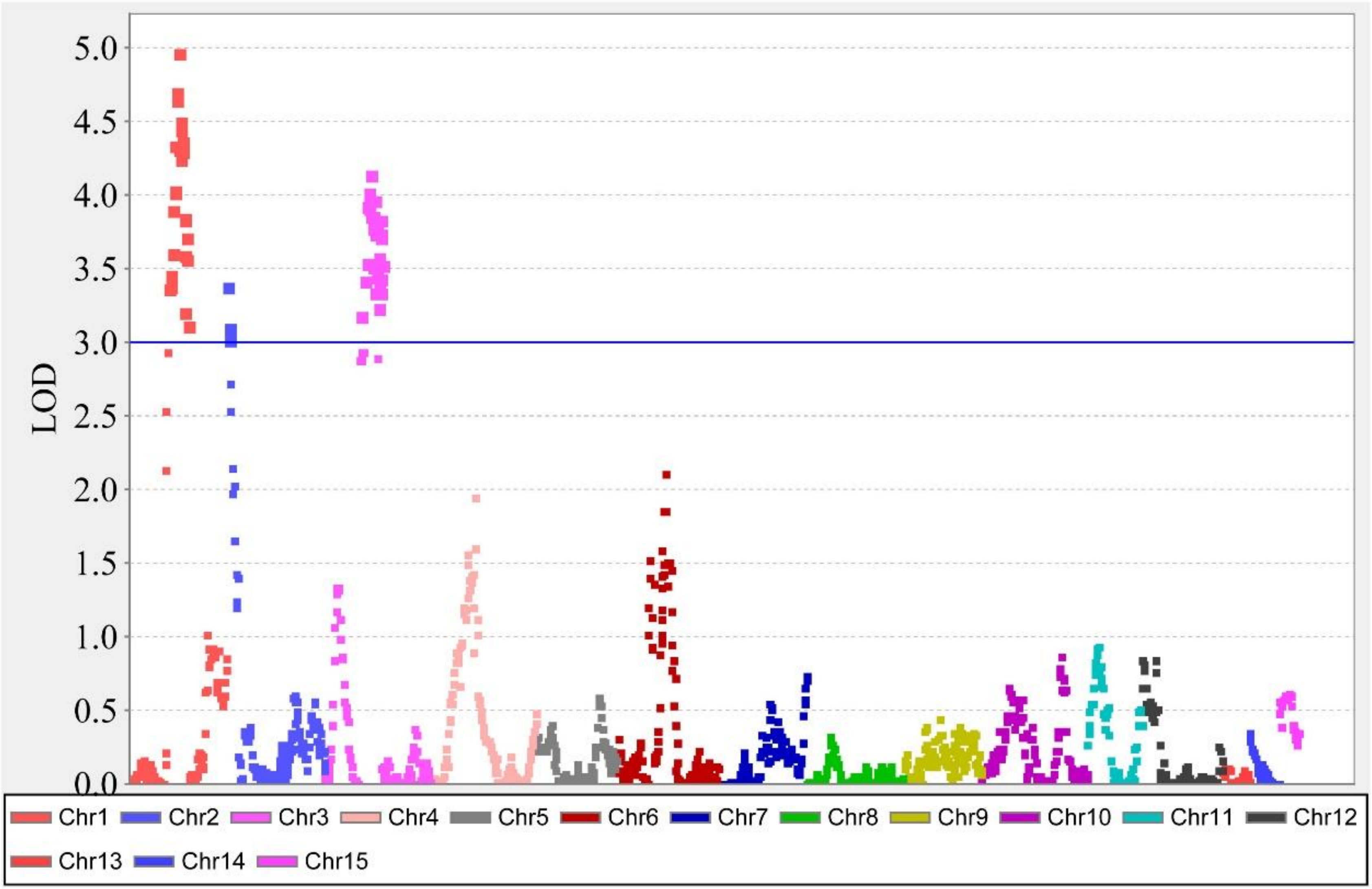

Figure S4. Manhattan plot of QTLs for mgrs. Based on the 1,000 permutation test, the LOD threshold for significant QTLs ($p < 0.05$) was 2.98, which was marked in a blue line.
